# Special Issue “Ovarian Cancer: Advances on Pathophysiology and Therapies”

**DOI:** 10.3390/ijms25105282

**Published:** 2024-05-13

**Authors:** Giovanni Tossetta, Annalisa Inversetti

**Affiliations:** 1 Department of Experimental and Clinical Medicine, Università Politecnica delle Marche, 60126 Ancona, Italy; 2 Department of Biomedical Sciences, Humanitas University, Via Rita Levi Montalcini 4, Pieve Emanuele, 20072 Milan, Italy; 3 IRCCS Humanitas Research Hospital, Via Manzoni 56, 20089 Rozzano, Italy

Ovarian cancer is a gynecologic cancer with a high mortality rate, and its incidence has increased significantly over the past 50 years. The high lethality of ovarian cancer, but also other types of cancer, is mainly due to its late diagnosis and to the occurrence of chemoresistance [1,2,3]. In fact, the occurrence of chemoresistance significantly worsens the outcome of this disease.

The evaluation of the expression of specific biomarkers led to important results for predicting the occurrence/progression of several cancerous [4,5,6,7,8,9,10] and non-cancerous [11,12,13] diseases, as well as identifying novel therapeutic targets [14,15,16,17,18,19].

However, despite advances in medical tumor therapy, the occurrence of chemoresistance and metastatic disease are the most common causes of death in patients with ovarian cancer [20,21]. Thus, new therapeutic approaches that can improve diagnosis and treatment outcomes are needed. To achieve this aim, we need a better understanding of the molecular changes occurring in ovarian cancer and the development of molecular biomarkers able to predict tumor behavior and the risk of disease recurrence and chemoresistance.

In this Special Issue, six articles and three reviews addressing the major problems in ovarian cancer pathophysiology and therapies have been selected for publication.

The study by Chacón and colleagues evaluated 112 coding genes upregulated in the aged nulliparous (NP) mouse ovary compared to the aged multiparous one as a control. Canonical gene ontology and pathway analyses showed a pro-oxidant state accompanied by an increased metabolism of inflammatory lipid mediators. Moreover, the authors found an upregulation of typical epithelial cell markers in the aged NP ovary during the pre-neoplastic phase of the mouse ovarian surface epithelial (MOSE) cell line. Thus, the aged NP ovary displays a multifaceted stress state resulting from oxidative imbalance and pro-inflammatory lipid signaling.

Zhu and colleagues assessed the presence of circulating tumor DNA in the blood (ctDNA) in women diagnosed with serous ovarian cancer and found tumor-specific variants (TSVs) in cancer cells. Moreover, post-surgery plasma showed the presence of ctDNA in 32% of the patients with visible residual disease, while 66% had no visible residual disease, and, of these, 77.4% of patients had detectable ctDNA.

In another study, Harbin and colleagues evaluated the *SYNE1* mutation frequency and effects in ovarian cancer and found that of 50 patients, 16 had a *SYNE1* mutation, and 15 had recurrent disease. The median TMB for *SYNE1*-mutated patients was 25 compared to 7 for *SYNE1*-wild-type patients. Moreover, gene expression related to immune cell trafficking, inflammatory response, and immune response was significantly increased in *SYNE1*-mutated patients.

The study by Alblihy and colleagues showed that MRE11 blockade by the inhibitor mirin induces synthetic lethality (SL) in BRCA2-deficient ovarian cancer cells compared to BRCA2-proficient controls. Moreover, increased cytotoxicity was associated with double-strand break accumulation, S-phase cell cycle arrest, and increased apoptosis. Furthermore, the authors found that mirin docks onto the active site of MRE11. MRE11 knockdown reduced cell viability in BRCA2-deficient ovarian cancer cells but not in BRCA2-proficient cells.

Finally, Chitcholtan and colleagues evaluated the anticancer effects of resveratrol, a natural compound with several beneficial effects [22,23,24,25], on ovarian cancer cell lines, i.e., OVCAR-8 and SKOV-3, grown on the chorioallantoic membrane (CAM) of chicken embryos. The authors found that resveratrol significantly reduced angiogenic activities, pNF-κB levels, and SLUG protein levels, suggesting that this compound may have the potential to impact the behavior of ovarian cancer progression.

In addition to these insightful research articles, two reviews in this Special Issue highlight the role of the solute carrier family 7 member 11 (SLC7A11) transporter (reviewed by Fantone and colleagues) and human epididymis protein 4 (HE4) (reviewed by Anastasi and colleagues) in ovarian cancer, two important players involved in ferroptosis modulation (SLC7A11) [26,27] and ovarian cancer detection (HE4) [28,29,30], respectively. Another review by Vorderbruggen and colleagues highlighted the role of proteolysis targeting chimeras (PROTACs) in ovarian cancer, reviewing the pharmacodynamic properties of these agents.

The nine articles published in this Special Issue prove the growing interest in finding new molecular targets and mechanisms involved in the diagnosis, pathogenesis, and treatment of ovarian cancer. We hope to provide our readers with a new representative and useful snapshot of the current problems in ovarian cancer pathophysiology in order to inspire new studies in this field. I personally acknowledge all the contributors to this Special Issue, the Editorial Board, and the assistant editors of the *International Journal of Molecular Sciences* for their support.
